# Medical Department of the University of Buffalo

**Published:** 1852-12

**Authors:** 


*Medical Department of the University of Buffalo.*—It will be borne in mind that the *regular term*, in this institution, will commence on the *fifth day of January*, 1853.

The college was opened for students desiring to pursue practical anatomy, on the first of November. Several classes were formed in the early part of that month, and dissections are now in progress under the supervision of the Demonstrator of Anatomy, Dr. H. B. Vandeventer. Instruction in clinical medicine and surgery is also given at the hospital during the preliminary term.

There is reason to expect an increased number of students at the regular session for the coming term.

The Museum is finished, adding greatly to the appearance, and convenience of the college building.

We conceive that we do not step beyond the limits of that delicacy which our connection with this institution should impose, when we say that, considering the opportunities for clinical teaching, and the facilities for prosecuting the different branches of medical education, Buffalo offers for the student advantages fully adequate to the most profitable expenditure of his time and attention during the usual period of collegiate instruction.

